# Ameliorating Racial Disparities in HIV Prevention via a Nurse-Led, AI-Enhanced Program for Pre-Exposure Prophylaxis Utilization Among Black Cisgender Women: Protocol for a Mixed Methods Study

**DOI:** 10.2196/59975

**Published:** 2024-08-13

**Authors:** Chen Zhang, Mitchell Wharton, Yu Liu

**Affiliations:** 1 School of Nursing University of Rochester Rochester, NY United States; 2 School of Dentistry and Medicine University of Rochester Rochester, NY United States

**Keywords:** artificial intelligence, PrEP care, PrEP, pre-exposure prophylaxis, nurse-led, AI, HIV prevention, HIV, prevention, AIDS, nurse, Black cisgender women, Black, cisgender, women, HIV pre-exposure prophylaxis, prophylaxis, biomedical, effectiveness, medical mistrust, Black women, nurse practitioners, chatbot, socioeconomic, HumanX technology, health care interventions

## Abstract

**Background:**

HIV pre-exposure prophylaxis (PrEP) is a critical biomedical strategy to prevent HIV transmission among cisgender women. Despite its proven effectiveness, Black cisgender women remain significantly underrepresented throughout the PrEP care continuum, facing barriers such as limited access to care, medical mistrust, and intersectional racial or HIV stigma. Addressing these disparities is vital to improving HIV prevention outcomes within this community. On the other hand, nurse practitioners (NPs) play a pivotal role in PrEP utilization but are underrepresented due to a lack of awareness, a lack of human resources, and insufficient support. Equipped with the rapid evolution of artificial intelligence (AI) and advanced large language models, chatbots effectively facilitate health care communication and linkage to care in various domains, including HIV prevention and PrEP care.

**Objective:**

Our study harnesses NPs’ holistic care capabilities and the power of AI through natural language processing algorithms, providing targeted, patient-centered facilitation for PrEP care. Our overarching goal is to create a nurse-led, stakeholder-inclusive, and AI-powered program to facilitate PrEP utilization among Black cisgender women, ultimately enhancing HIV prevention efforts in this vulnerable group in 3 phases. This project aims to mitigate health disparities and advance innovative, technology-based solutions.

**Methods:**

The study uses a mixed methods design involving semistructured interviews with key stakeholders, including 50 PrEP-eligible Black women, 10 NPs, and a community advisory board representing various socioeconomic backgrounds. The AI-powered chatbot is developed using HumanX technology and SmartBot360’s Health Insurance Portability and Accountability Act–compliant framework to ensure data privacy and security. The study spans 18 months and consists of 3 phases: exploration, development, and evaluation.

**Results:**

As of May 2024, the institutional review board protocol for phase 1 has been approved. We plan to start recruitment for Black cisgender women and NPs in September 2024, with the aim to collect information to understand their preferences regarding chatbot development. While institutional review board approval for phases 2 and 3 is still in progress, we have made significant strides in networking for participant recruitment. We plan to conduct data collection soon, and further updates on the recruitment and data collection progress will be provided as the study advances.

**Conclusions:**

The AI-powered chatbot offers a novel approach to improving PrEP care utilization among Black cisgender women, with opportunities to reduce barriers to care and facilitate a stigma-free environment. However, challenges remain regarding health equity and the digital divide, emphasizing the need for culturally competent design and robust data privacy protocols. The implications of this study extend beyond PrEP care, presenting a scalable model that can address broader health disparities.

**International Registered Report Identifier (IRRID):**

PRR1-10.2196/59975

## Introduction

HIV pre-exposure prophylaxis (PrEP) is a safe and proven biomedical strategy to prevent HIV transmission among cisgender women [1-3]. However, Black cisgender women remain underrepresented throughout the PrEP care continuum compared to their White counterparts [4,5]. Our preliminary studies reveal that multilevel barriers, such as a lack of PrEP awareness and knowledge, low perceived risk, limited access to care, medical mistrust, and intersectional racial or HIV stigma, often hinder PrEP utilization [6-8]. Conversely, the “PrEP care cascade” model describes the progressive stages of PrEP care [9]. It suggests that the engagement of providers and patients is critical to optimal PrEP care implementation. Research has demonstrated that nurse practitioners (NPs) are well suited to lead PrEP care initiatives [8,10]. Our recent meta-analysis showed that NPs reported a 40% higher likelihood than physicians to prescribe PrEP and provide PrEP care to patients with indications [8].

Furthermore, evidence shows that an NP-based PrEP care model may successfully engage and retain patients in PrEP care [10]. NPs’ holistic care approach integrates patients’ psychological and social factors with biological components, increases patients’ satisfaction in health care, and is a strong predictor of retaining and adhering to treatment and prevention care plans [7,8,10]. Therefore, involving NPs is critical when developing programs to facilitate PrEP utilization. Despite these benefits, interventions promoting PrEP utilization involving NPs are scarce [8,10]. Our preliminary studies showed that a lack of awareness, insufficient clinical support, and a lack of workforce all contributed to NPs’ under-representativeness in PrEP care [7,8].

Despite digital technology-assisted HIV prevention programs being effective and well received among Black cisgender women [11,12], they are severely underrepresented in relevant research [13-15]. Equipped with the rapid evolution of artificial intelligence (AI) and advanced large language models, chatbots effectively facilitated health care communication and linkage to care in various domains [13,16,17]. In the HIV-related field, chatbots can provide personalized health information, remind patients to take their medications, schedule appointments, and offer psychological support. They can also assist in triaging patients, identifying high-risk patients, and providing tailored interventions to improve PrEP adherence and retention in care [14,15]. Chatbots can provide round-the-clock access to PrEP information, dispel myths, and reduce stigma by offering anonymous support, which is crucial in overcoming barriers such as medical mistrust and low perceived risk [14,15,18-20]. While our preliminary studies show high acceptability for chatbot technology among Black cisgender women, its full potential in addressing PrEP disparities in this population remains untapped [15,17,20,21]. By integrating chatbot technology into PrEP care models, especially those led by NPs, we can enhance PrEP awareness, improve patient engagement, and ultimately increase PrEP utilization among Black cisgender women.

Therefore, our study harnesses NPs’ holistic care capabilities and the power of AI through natural language processing algorithms, providing targeted, patient-centered facilitation to PrEP care. Our overarching goal is to create a nurse-led, stakeholder-inclusive, and AI-powered program to facilitate PrEP utilization among Black cisgender women, ultimately enhancing HIV prevention efforts in this vulnerable group [8,10,22,23]. This objective will be achieved through the following 3 phases. Phase 1 aims to understand the preferences and needs of Black cisgender women and NPs for the development of a culturally responsive, AI-powered chatbot designed to facilitate PrEP care. Phase 2 aims to develop and refine a tailored, culturally appropriate, theory-guided, and AI-powered chatbot platform using a participatory design approach. Phase 3 aims to evaluate the developed chatbot’s acceptability, feasibility, and effectiveness in facilitating linkage to PrEP among Black cisgender women. This project aims to mitigate health disparities and advance innovative, technology-based solutions.

## Methods

### Study Populations and Inclusion Criteria

Using our well-established research infrastructure, we will recruit key stakeholders nationwide who may contribute to understanding AI-powered chatbots in the HIV care and prevention continuum using semistructured in-depth interviews. These key stakeholders include PrEP-eligible Black cisgender women (n=50: n=10, 20% in phase 1; n=20, 40% in phase 2; and n=20, 40% in phase 3) and NPs who provide HIV treatment and prevention services to patients (n=10). We will use semistructured interviews to elicit information regarding these stakeholders’ opinions on chatbot development and evaluation. Black cisgender women who participate in phase 1 will not be invited to phases 2 and 3 to avoid the issue of information contamination. A community advisory board (CAB) with volunteer members (n=10) representing key stakeholders will be established to ensure our research reflects the priorities of the study population. These members will be selected to represent different characteristics (eg, socioeconomic status and geolocations) in the targeted community. Leaders from local community organizations and general community members will also be invited to strengthen the acceptability and quality of the proposed research. Once the CAB members are recruited, they will remain throughout the study.

For *PrEP-eligible* *Black cisgender women*, we will recruit 50 Black cisgender women who are eligible for HIV prevention services. The inclusion criteria include (1) aged 18 years or older, (2) self-identifying as Black or African American, (3) self-reported as female, (4) not tested as HIV positive (either HIV negative or unknown status), (5) eligible for HIV prevention (engaging in unprotected sex, having multiple sexual partners, or history of sharing needles with others), (6) willing to provide consent for participation, (7) fluent in English, and (8) able to engage in either in-person or web-based interviews. For *HIV care providers*, we will recruit 10 NPs who provide HIV prevention or treatment to patients. Eligible health providers need to (1) be aged 18 years or older, (2) work as a nurse or NP, (3) have ever provided HIV prevention or treatment in the past 12 months, (4) be willing to provide consent for participation, (5) be fluent in English, and (6) be able to engage in either in-person or web-based interviews. For *CAB members*, we will invite 10 members representing different characteristics (eg, socioeconomic status and geolocations) in the targeted community. Eligible CAB members need to be (1) aged 18 years or older, (2) interested in HIV prevention and treatment topics, (3) fluent in English, (4) willing to provide consent for participation, and (5) able to engage in either in-person or web-based meetings.

### Recruitment Strategy

Participants will be recruited via multiple routes*.* For *HIV nurses or NPs*, an institutional review board (IRB)–approved recruitment message and flyers will be distributed via local newsletters or the listserve among members of the Association of Nurses in AIDS Care or the National Black Nurses Association using the IRB-approved recruitment email script message for nurses. We will recruit *CAB members* via email listserves and flyers for recruitment. For *PrEP-eligible* *Black cisgender women*, we will distribute the recruitment information via the regional ResearchMatch portal to recruit willing volunteers. Anyone interested in this study can use the survey link or scan the QR code for eligibility screening. They can also contact the research team members via email or phone, and the research team will direct them to the screening procedures. Other interested participants will be recruited using “snowball” chain referral sampling, where we ask Black cisgender women, nurses, or potential CAB members to identify others within their networks who might be interested in the study. Participants can either complete the brief screening over the phone or be emailed a link to complete the screening via REDCap (Research Electronic Data Capture; Vanderbilt University). A referrer will be compensated a small amount (ie, US $5) for each peer they successfully refer, with a cap on referral payments at 3 successful enrollments from participants. When potential participants contact us, the study team members will explain the study and obtain verbal consent to proceed with phone screening using the script, or they can access the REDCap link directly from the study flyer by scanning the QR code.

### Description of the Chatbot

#### Overview

Our project leverages the cutting-edge HumanX chatbot technology developed by KLaunch [24], which is designed to integrate conversational AI within businesses and research. This platform, combined with Smartbot360’s secure chatbot technology, offers a seamless, safe, and highly personalized communication experience aimed at facilitating PrEP utilization among Black cisgender women at risk of HIV infection. The integrated system ensures HIPAA (Health Insurance Portability and Accountability Act) compliance, safeguarding protected health information (PHI) through technical, physical, and administrative measures.

#### Security and Compliance

The HumanX and Smartbot360 platforms prioritize protecting patient information, ensuring that all data, including PHI, are encrypted in transit and at rest. Robust access controls and authentication measures prevent unauthorized access, and regular security assessments address evolving threats and vulnerabilities. Comprehensive risk assessments tailored to the health care context help identify potential risks to PHI and implement mitigating strategies to ensure ongoing HIPAA compliance. Our dynamic compliance strategy adapts to regulatory changes and incorporates best practices in data protection, with all personnel trained on HIPAA requirements.

#### Third-Party Assessments

Independent auditors thoroughly evaluate the SmartBot360 and HumanX technologies to ensure they meet or exceed HIPAA compliance standards. These third-party assessments validate our security measures, risk management practices, and compliance with health care regulations, offering our stakeholders an additional layer of assurance.

### Study Phases

In this proposed study, our primary objectives will be achieved through the following 3 phases within the 18-month timeframe by recruiting 50 Black cisgender women and 10 NPs.

#### Phase 1: Exploration (Months 1-3)

Phase 1 of the proposed study aims to understand preferences for the development of a nurse-led, stakeholder-inclusive, and AI-powered program to facilitate PrEP utilization among Black cisgender women, ultimately enhancing HIV prevention efforts in this group. Research indicates that adopting the participatory design [23,25-27] is feasible by actively engaging stakeholders within target communities, consequently providing a solid foundation for the newly developed tools. Thus, our project will use a stakeholder-inclusive and participatory design approach [25-27] to collect critical stakeholders’ opinions and feedback. Our design will also incorporate culture-oriented content expressly for Black cisgender women by consulting a CAB, PrEP-eligible women, and NPs who provide HIV prevention and treatment services. Using in-depth interviews with Black cisgender women and NPs, we will gather their opinions regarding the chatbot. By the end of phase 1 of this study, we expect an enhanced understanding of factors affecting PrEP utilization among Black cisgender women and preferences regarding the chatbot prototype in the later phases.

#### Primary Outcomes and Assessment for Phase 1

We will use a stakeholder-inclusive and participatory design approach to evaluate two primary outcomes: (1) stakeholder preferences for the chatbot development will be assessed through in-depth interviews with Black cisgender women and NPs—we will gather opinions on features, content, and cultural appropriateness; and (2) factors influencing PrEP utilization among Black cisgender women will be assessed through thematic analysis of interview transcripts.

#### Phase 2: Development (Months 4-8)

We will develop and refine a tailored and culturally appropriate, theory-guided, and AI-powered chatbot platform using participatory design to ensure cultural appropriateness for the target population. The basic structure of chatbot scripts (eg, PrEP facts and HIV risks) will be extracted from the Centers for Disease Control and Preventions (CDC) and other health departments [23,25,26] and validated studies [20,27]. We will develop features (ie, complexity and aesthetics) and workflow (ie, frequency of the interaction) of the platform that best meets end-users needs [28-31]. Once the initial platform is developed, its features will be tested within 2 small groups of eligible Black cisgender women who did not participate in phase-1 interviews. We will use the think-aloud strategy in the refinement process. The first 10 eligible Black cisgender women will interact with the chatbot. We will use their feedback to improve the chatbot and launch it with another 10 Black cisgender women. After collecting input from the second group, we will further enhance the chatbot. A total of 20 Black cisgender women will be recruited in phase 2. Before launching the chatbot, we will provide a training session to the NPs to guide them in providing psychological and clinical support to women during their interactions with the chatbot, particularly when women have unaddressed needs.

#### Primary Outcomes and Assessment for Phase 2

We will develop and refine a tailored, culturally appropriate, theory-guided, and AI-powered chatbot platform using participatory design to ensure cultural appropriateness for the target population with two primary outcomes: (1) chatbot development—the initial development of the chatbot platform, including scripts and features, will be based on CDC guidelines and validated studies; and (2) user feedback—the usability and functionality of the chatbot will be assessed through think-aloud sessions with 2 small groups of Black cisgender women (10 in each group) who will interact with the chatbot and provide feedback.

#### Phase 3: Evaluation (Months 9-18)

We aim to evaluate and improve the developed chatbot in phase 3. We will assess the chatbot’s acceptability and feasibility in facilitating linkage to PrEP among another 20 eligible Black cisgender women. Using the longitudinal mixed methods, we will determine the feasibility (Feasibility Scale for assessment) [32], acceptability (Evidence-Based Practice Attitude Scale) [33], and user experiences (Usability Metric for User Experience Scale) [34] of the chatbot at baseline (eg, chatbot initiation), 3 months, and 6 months using both surveys and in-depth interviews. By triangulating the collected quantitative and qualitative data, we will enhance the chatbot to better address the needs of the target population in a real-world setting. We will also conduct a focus group among the 10 NPs who deliver the holistic approach to explore their feedback on the effectiveness of the chatbot platform. We will continuously improve the chatbot throughout the study period with the collected information.

#### Primary Outcomes and Assessment for Phase 3

We will evaluate and improve the developed chatbot using four leading indicators: (1) the acceptability of the chatbot will be measured using the Evidence-Based Practice Attitude Scale [29] at baseline, 3 months, and 6 months; (2) the feasibility of the chatbot will be assessed using the Feasibility Scale [30] for assessment; (3) user experience will be evaluated using the Usability Metric for User Experience Scale [31]; and (4) effectiveness in facilitating linkage to PrEP will be assessed through surveys and in-depth interviews with another 20 eligible Black cisgender women.

This proposed study will conduct all research activities on the web or at university locations. Based on our well-established research infrastructure, we will recruit 50 eligible Black cisgender women (n=10, 20% in phase 1; n=20, 40% in phase 2; and n=20, 40% in phase 3) and 10 NPs. Study information will be distributed through community events, health clinics, professional organizations, and social media. If a participant drops out from any phase, a replacement will be recruited to maintain the sample size. To ensure the feasibility of recruitment, we will use our extensive network of community partners and leverage our previous success in similar recruitment efforts.

Additionally, recruitment progress will be regularly monitored, and strategies will be adjusted to meet the target sample size within the study timeline. During the chatbot development and refinement, monthly meetings with CAB members will be held for feedback. A project start-up meeting, including training sessions for team members, will be conducted before data collection. Our interdisciplinary team is uniquely equipped to carry out this innovative project.

### Data Analytical Plan

*Quantitative data* include demographic information, socioeconomic characteristics, and implementation and usability outcomes. Due to the limited sample size (n=20), we will perform descriptive analyses to present participants’ characteristics (eg, demographics, sexual behaviors, and HIV risks) and user experiences (eg, feasibility and acceptability) to observe potential similarities and differences in particular characteristics’ implementation and use outcomes (eg, age group). *Qualitative data* (ie, opinions regarding the chatbot development and experience) will be transcribed and loaded into qualitative analytical software for analysis. This will follow the 5-step framework analysis approach, which includes familiarization (describing potential themes), thematic framework (generating a nascent code or subcode list), indexing (producing a final consensus code list), charting (organizing critical thematic constructs into hierarchical themes), and mapping and interpretation (summarizing and interpreting) [32]. Participants’ verbal utterances collected by think-aloud sessions will be transcribed and analyzed by propositional analysis (an analysis to create a basic semantic model of the text to comprehend an individual’s thoughts and critical ideas for a given product) [33]. *Usability data* gathered by the dashboard will be used to describe user activities and profiles, such as log-in time, bounce rate, retention rate, and conversation channels. *Conversation data* will be downloaded from the dashboard and explored using the systematic thematic analysis strategies (familiarization, code assignment, pattern identification, and defining and reporting themes) [34-36]. Specifically for data collected from the NPs, we will analyze both quantitative and qualitative responses. Quantitative data will include NPs’ demographic information, professional background, and their engagement with PrEP care. We will use descriptive statistics to summarize these data and examine correlations between NP characteristics and their effectiveness in promoting PrEP utilization. Qualitative feedback from NPs on the chatbot’s functionality, its integration into their workflow, and its impact on patient engagement will be transcribed and analyzed using the same 5-step framework analysis approach mentioned earlier. This analysis will help us identify specific barriers and facilitators NPs face in utilizing the chatbot for PrEP care and how the chatbot can be optimized to support their efforts. By combining these methods, we aim to provide a comprehensive understanding of the chatbot’s usability, effectiveness, and potential areas for improvement, particularly in facilitating PrEP utilization among Black cisgender women and supporting NPs in their role.

### Ethical Considerations

Upon submitting this manuscript, we obtained IRB approval for the research activities of recruiting participants to gather their preferences of chatbot features (University of Rochester Research Subjects Review Board: STUDY00009105).

All participants will provide informed consent before participating in the study. The consent process will be conducted on the web via REDCap, a secure web-based platform designed for research data collection and management. An information sheet detailing the study’s purpose, procedures, risks, and benefits will be provided to participants prior to study participation. Consent will be documented through an electronic consent form that participants will complete before any study activities begin.

Data collected during the study will be anonymized and deidentified to protect participants’ privacy. All data will be stored securely on the university’s servers, and access will be restricted to authorized study team members only. Measures such as encryption, secure servers, and compliance with HIPAA regulations will be implemented to safeguard participant information. Audio recordings of interviews will be transcribed and deidentified before analysis, and all identifiers will be deleted once transcription is complete.

Participants will be compensated for their time and contributions to the study. Black cisgender women participating in the in-depth interviews will receive US $30 each, while CAB members will receive US $20 for each meeting, for a total of 3 times throughout the study. Additionally, a small incentive of US $5 will be provided for each successful referral made through snowball sampling, with a cap on referral payments at 3 successful enrollments. Compensation will be provided irrespective of the completion status of the interviews or activities to ensure fairness.

## Results

As of August 8, 2024, our project is in the initial stages of implementation. The IRB protocol for phase 1 was approved in May 2024. We plan to start recruitment for Black cisgender women and NPs in September 2024, with the aim to collect information to understand their preferences regarding chatbot development. We are currently working on attaining IRB approval for phases 2 and 3. To date, we have made significant progress in networking for participant recruitment, and we have established connections with individuals and colleagues who have expressed interest in assisting with the recruitment process, leveraging our established network and infrastructure. This step is instrumental in ensuring a robust and diverse participant base, aligning with our study’s objective to engage Black cisgender women in PrEP care. We plan to collect data soon, and further updates on the progress of recruitment and data collection will be provided as the study advances.

## Discussion

### Expected Findings

Our proposed study harnesses AI technology and nursing skills through an AI-powered chatbot designed to facilitate PrEP care utilization among Black cisgender women. This culturally responsive, nurse-led, and AI-powered chatbot encapsulates biomedical, psychological, and sociocultural factors affecting PrEP utilization, making it a holistic tool for HIV prevention. Our model is scalable both geographically and technologically, making it capable of addressing health disparities beyond the immediate context of this study. As AI improves with increased data and user engagement, we anticipate the AI chatbot will significantly improve PrEP awareness, adherence, and retention among Black cisgender women by providing personalized, accessible, and stigma-free support.

### Comparison to Prior Work

Implementing AI-powered chatbots in health care has shown promise in various domains, including mental health support, chronic disease management, and patient education [13-15]. Previous studies have demonstrated that digital technology-assisted health care can improve patient engagement and outcomes [13-15]. However, few studies have specifically focused on using AI chatbots to address HIV prevention among Black cisgender women [11,12]. Our study builds on existing research by integrating cultural competence and stakeholder feedback into the chatbot design, making it uniquely tailored to the needs of this population. Our approach offers a novel solution by combining AI technology with nursing expertise, bridging the gap between digital health innovations and culturally sensitive care.

### Limitations and Strengths of the Protocol

Implementing AI-powered chatbots presents several challenges and strengths [24,37,38]. Expected limitations include the following. First, technology accessibility: PrEP-eligible individuals without consistent access to technology or who are less comfortable with digital tools may face unintended exclusion, potentially reinforcing existing health care disparities. Second, data privacy: ethical considerations regarding data privacy and security are critical, as chatbots gather sensitive health information. Ensuring HIPAA compliance, robust encryption, and clear communication about data use is essential to maintain users’ trust. Third, generalizability: while our study focuses on Black cisgender women, the findings may not be generalizable to other populations without further adaptation and testing. On the other hand, there are several strengths identified in the proposed study. First, cultural competence: our chatbot is designed to meet the unique needs of Black cisgender women by incorporating their cultural contexts and addressing their specific concerns, enhancing its relevance and effectiveness. Second, scalability: the model is scalable both geographically and technologically, making it capable of addressing health disparities beyond the immediate context of this study. Third, holistic approach: the integration of biomedical, psychological, and sociocultural factors provides a comprehensive approach to HIV prevention, potentially leading to better health outcomes.

### Conclusion

As the AI component evolves with more data and user interaction, we foresee opportunities for further technological advancements, enhancing the efficacy of our model. Upon completing this pilot, we aim to roll out this innovative program to other marginalized populations to expand its reach and impact using a subsequent large-scale hybrid type I study design. By doing so, we aim to contribute tangible, scalable solutions to the pressing challenge of racial disparities in HIV prevention. This vision aligns seamlessly with the funding foundation’s key priorities, including challenging conventional strategies, demonstrating potential as a best-in-class intervention, narrowing gaps in health equity, and showcasing the potential for scalability. We are deeply committed to realizing this vision and driving forward the fight against racial disparities in HIV prevention and beyond.

## Abbreviations

AI: artificial intelligence
CAB: community advisory board
CDC: Centers for Disease Control and Prevention
HIPAA: Health Insurance Portability and Accountability Act
IRB: institutional review board
NP: nurse practitioners
PHI: protected health information
PrEP: pre-exposure prophylaxis
REDCap: Research Electronic Data Capture
